# When Teratoma Masquerades: A Rare Case of Intrapleural Mature Cystic Teratoma Mimicking Tuberculous Empyema

**DOI:** 10.1002/rcr2.70252

**Published:** 2025-06-18

**Authors:** Nik Nuratiqah Nik Abeed, Ng Boon Hau, Nor Safiqah Sharil, Andrea Ban Yu Lin, Marfua'h Nik Ezzamudin, Hairulfaizi Haron

**Affiliations:** ^1^ Respiratory Unit, Faculty of Medicine Universiti Kebangsaan Malaysia Cheras Malaysia; ^2^ Faculty of Medicine and Health Sciences Universiti Sains Islam Antarabangsa Kuala Lumpur Malaysia; ^3^ Oncology Department, Faculty of Medicine Universiti Kebangsaan Malaysia Cheras Malaysia; ^4^ Cardiothoracic Unit, Faculty of Medicine Universiti Kebangsaan Malaysia Cheras Malaysia

**Keywords:** cystic, intrapleural, mature, teratoma

## Abstract

Teratomas are germ cell tumours generally gonadal in origin and very rare, arising from extra gonadal tissue. The most common extragonadal teratomas are mediastinal, and the majority are benign. We report a case of a 49‐year‐old lady with recurrent right complex pleural effusion occurring a month after the completion of treatment for tuberculous empyema. Imaging from ultrasound of the thorax and contrasted enhanced computed tomography (CECT) thorax revealed multiseptated and loculated effusion in the thorax without any mediastinal and lung involvement. Surprisingly, pleural fluids were negative for tuberculosis. Despite chest drainage and initial treatment for bacterial empyema without improvement, she underwent video assisted thoracoscopy and decortication of the right pleura, and histopathological analysis revealed a mature cystic teratoma. She was discharged in good health and under yearly surveillance. The rarity of intrapleural mature cystic teratoma and its misleading presentation due to the concurrent tuberculous empyema make this case noteworthy.

## Graphical abstract

We report a case of a 49‐year‐old lady with recurrent right complex pleural effusion occurring a month after completed treatment of tuberculous empyema. Imaging and diagnostic work‐up revealed a mature cystic teratoma. The rarity of intrapleural mature cystic teratoma and its misleading presentation due to the concurrent tuberculous empyema makes this case noteworthy.

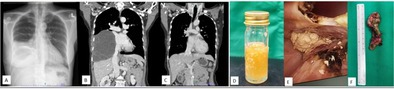

## 1 Introduction

A benign cystic mature teratoma originating from the pleural space without mediastinal involvement is extremely rare. Most teratomas grow slowly, with patients typically remaining asymptomatic. However, in this case, the presence of a concurrent tuberculous infection exacerbated symptoms, prompting the patient to seek medical attention. Tuberculous empyema rarely recurs within a short period unless there is medication non‐compliance or misdiagnosis. This case underscores the importance of considering alternative diagnoses in cases of recurrent empyema or complex pleural effusion, especially when clinical responses are poor and no obvious primary lesion is identified.

## 2 Case Report

A 49‐year‐old woman, diagnosed with right tuberculous empyema, presented with fever, shortness of breath and a 2‐kg weight loss over 3 weeks. Clinically, she had right pleural effusion. A chest radiograph and bedside thoracic ultrasound confirmed loculated right pleural effusion. Blood tests showed leukocytosis with normal renal and liver function. A right chest drain was inserted, showing purulent exudative fluid. *Mycobacterium tuberculosis* Polymerase Chain Reaction (PCR) was positive, although results were negative for acid‐fast bacilli (AFB), mycobacterial culture and cytology.

Due to loculated empyema, she received fibrinolytic therapy with low‐dose intrapleural alteplase of 5 mg; however, this was complicated by bleeding at the chest drain site, preventing further cycles of fibrinolysis. She was started on a nine‐month course of anti‐tuberculous treatment with Akurit‐4 according to the body weight and showed clinical and radiological improvement by the end of therapy.

One month post completed treatment, she developed recurrent complex right pleural effusion. Imaging via thoracic ultrasound and contrast‐enhanced computed tomography (CECT) revealed a recurrent, multiseptated, loculated right pleural effusion. A chest drain was reinserted, and pleural fluid analysis showed straw‐coloured, exudative fluid with a high lactate dehydrogenase (LDH) level of 469 U/L, though bacterial culture and tuberculosis including Xpert Mycobacterium/Rifampicin (MTB/RIF) tests were negative. Blood parameters indicated an inflammatory response, with elevated white blood cells (13 × 10^9^/L) and C‐reactive protein (5.41 g/dL), but blood cultures were negative.

**FIGURE 1:**
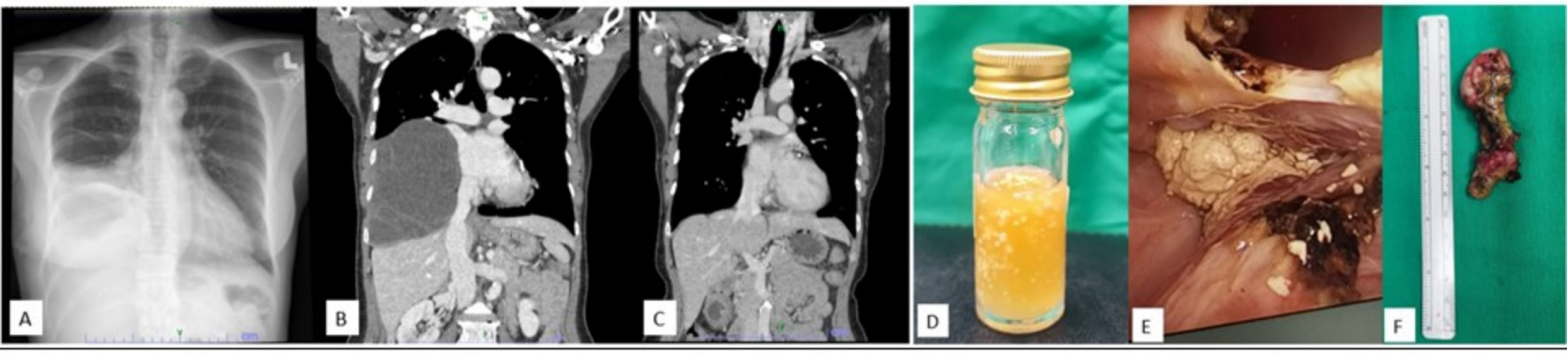
(A) Chest radiograph showed right moderate pleural effusion; (B) CECT coronal view showed right pleural effusion; (C) CECT surveillance post decortication showed resolving right pleural effusion; (D) Pleural fluid showed proteinaceous yellowish fluid; (E) Cheesy mass embedded in parietal pleural via video assisted thoracoscopy; (F) Pleural tissue with the tumour 8 cm length.

She was treated for bacterial empyema with a 4‐week course of piperacillin‐tazobactam, but her condition did not improve. The patient was then referred to a cardiothoracic surgeon for decortication. During video‐assisted thoracoscopic surgery (VATS), surgeons found multiloculated thickened pleural cortex adherent to the pericardium and diaphragm, with Ghon complexes and 1 L of proteinaceous fluid (Figure 1) surprisingly, pleural fluid and tissue tested negative for tuberculosis, and pleural tissue histopathological examination showed macroscopically multiloculated cysts containing yellowish pasty contents. The cyst is composed of mature ectodermal, mesodermal and endodermal components such as keratinized stratified squamous epithelium, adnexa sweat glands, sheets of adipose tissue, smooth muscle bundles, gastric foveolar epithelium, ciliated respiratory and tubal epithelium, renal tubular epithelium and endocervical epithelium revealed mature cystic teratoma.

Postoperatively, the patient was discharged in good health and placed under annual surveillance with CECT thorax. After 3 years of follow‐up, she remains asymptomatic and radiologically stable.

## 3 Discussion

Mature cystic teratoma is a benign tumour originated from gonads. Tumour can occur in extragonadal regions, especially the mediastinum. However, intrapleural mature cystic teratoma without mediastinal involvement is extremely rare.

The patients are often asymptomatic and the tumour is discovered incidentally on chest radiographs obtained for other reasons. To our knowledge, this is the third case reported. The first case reported a young lady with acute chest pain and shortness of breath; and the CT showed a complex cystic mass within the left pleural cavity. The patient did thoracotomy with removal of the cystic mass, with confirmed mature cystic teratoma [1]. The second case was empirically treated as bacterial empyema given fibrinolytic therapy but poor response; hence, it did decortication, with intraoperative revealed hair‐like structures in the purulent fluid [2]. Otherwise, Katoto et al. reviewed 63 cases of mature teratoma presented with pleural effusion, with the origin of the teratoma being mediastinal [3].

Similar to other cases reported, the majority of the cases were misdiagnosed as tuberculous or bacterial empyema and remaining symptomatic warranted further investigation. This case is noteworthy in terms of atypical presentation, intrapleural in origin without mediastinal involvement and its misleading presentation due to the concurrent tuberculous empyema. The treatment of tuberculous empyema with fibrinolytic therapy and anti‐tuberculous therapy without recovery leading to decortication is the best option.

## Author Contributions

Nik Nuratiqah Nik Abeed as author constructing the case. Ng Boon Hau and Nor Safiqah Sharil contribute to discussion alignment. Andrea Ban Yu Lin is involved in the idea of the discussion. Marfu'ah Nik Ezzamudin contributes to writing a follow‐up care and continuation of care as an oncologist. Hairulfaizi Haron contributes to providing the picture during surgical intervention.

## Ethics Statement

The authors declare that written informed consent was obtained for the publication of this manuscript and accompanying images using the consent form provided by the Journal.

## Conflicts of Interest

A.B.Y.L. is an Editorial Board member of Respirology Case Reports and a co‐author of this article. She was excluded from all editorial decision‐making related to the acceptance of this article for publication. The other authors have no conflicts of interest to declare.

## Data Availability

Research data are not shared.
